# Tailoring the Stabilization and Pyrolysis Processes of Carbon Molecular Sieve Membrane Derived from Polyacrylonitrile for Ethylene/Ethane Separation

**DOI:** 10.3390/membranes12010093

**Published:** 2022-01-15

**Authors:** DaeHun Kim, YongSung Kwon, Jung-Hyun Lee, Seong-Joong Kim, You-In Park

**Affiliations:** 1 Green Carbon Research Center, Korea Research Institute of Chemical Technology (KRICT), Gajeong-ro 141, Yuseong-gu, Daejeon 34114, Korea; daehun72@krict.re.kr (D.K.); yskwon@krict.re.kr (Y.K.); 2 Department of Chemical and Biological Engineering, Korea University, 5-1 Anam-dong, Seongbuk-gu, Seoul 136-713, Korea; leejhyyy@korea.ac.kr; 3 Department of Chemical and Biomolecular Engineering, Korea Advanced Institute of Science and Technology (KAIST), Daejeon 34141, Korea; 4 Center for Convergence Bioceramic Materials, Convergence R&D Division, Korea Institute of Ceramic Engineering and Technology (KICET), 202 Osongsaengmyeong 1-ro, Osong-eup, Heungdeok-gu, Cheongju-si 28160, Chungcheongbuk-do, Korea

**Keywords:** olefin/paraffin, carbon molecular sieve membrane, polyacrylonitrile, stabilization, pyrolysis

## Abstract

For ethylene/ethane separation, a CMS (carbon molecular sieve) membrane was developed with a PAN (polyacrylonitrile) polymer precursor on an alumina support. To provide an excellent thermal property to PAN precursor prior to the pyrolysis, the stabilization as a pre-treatment process was carried out. Tuning the stabilization condition was very important to successfully preparing the CMS membrane derived from the PAN precursor. The stabilization and pyrolysis processes for the PAN precursor were finely tuned, and optimized in terms of stabilization temperature and time, as well as pyrolysis temperature, heating rate, and soaking time. The PAN stabilized at >250 °C showed improved thermal stability and carbon yield. The CMS membrane derived from stabilized PAN showed reasonable separation performance for ethylene permeance (0.71 GPU) and ethylene/ethane selectivity (7.62), respectively. Increasing the pyrolysis temperature and soaking time gave rise to an increase in the gas permeance, and a reduction in the membrane selectivity. This trend was opposite to that for the CMS membranes derived from other polymer precursors. The optimized separation performance (ethylene permeance of 2.97 GPU and ethylene/ethane selectivity of 7.25) could be achieved at the pyrolysis temperature of 650 °C with a soaking time of 1 h. The separation performance of the CMS membrane derived from the PAN precursor was comparable to that of other polymer precursors, and surpassed them regarding the upper bound trade off.

## 1. Introduction

The olefin/paraffin separation using a conventional distillation process requires high energy consumption due to their similar physical properties [1,2,3]. Therefore, to replace or support the energy-intensive distillation process, many researchers have studied membrane technology to provide a cost-effective and relatively simple separation process [4,5,6]. In particular, a carbon molecular sieve membrane has shown excellent olefin/paraffin separation performance, which was attributed to the rigid pore structure [7]. The rigid slit-like pore structure of a CMS membrane favors the diffusion of slimmer olefin over paraffin [8,9]. Thus, a CMS membrane can achieve excellent selectivity by molecular sieving separation in its ultra-micropores (4–5 Å), whereas the sorption property in the micropores (6–20 Å) can provide high gas permeability [10,11,12]. Furthermore, the CMS membrane does not undergo plasticization, unlike polymeric membranes. This is due to the rigid pore structure, and leads to no change in physical properties [13,14]. Therefore, the excellent separation performance of a CMS membrane can be used in harsh environments. 

A variety of polymer precursors, such as polyacrylonitrile (PAN) [15,16], poly furfuryl alcohol (PFA) [17,18], polyimide [19,20], phenolic resin [21,22], poly phenylene oxide (PPO) [23,24], cellulose (CA) [25], and polymers of intrinsic microporosity (PIM) [26,27], have been studied for use as CMS membranes [3]. In particular, for olefin/paraffin separation, polyimide precursors have commonly been employed due to their high free volume and excellent thermal stability, providing both high permeability and selectivity. The CMS membrane derived from Matrimid resulted in an ethylene permeability of 8.3–18.7 barrer, and ethylene/ethane selectivity of 6.31–12.3 [8,28]. On the other hand, the bulky CF_3_ group of 6FDA-based polyimides provides low chain-packing density and high fractional free volume, which gives rise to higher gas permeability of a CMS membrane. Moreover, 6FDA-based polyimides are favorable for tuning their chemical structures, which offers a variety of physical properties. For these reasons, many researchers have developed CMS membranes with a variety of 6FDA-based polyimides, such as 6FDA-DAM, 6FDA-DABA, 6FDA/BPDA-DAM, and 6FDA/DETDA-DABA. The CMS membranes derived from 6FDA-based polyimide showed ethylene permeability of 10–58.7 barrer, and ethylene/ethane selectivity of 3.9–11 [29,30,31,32]. PIM-based CMS membranes are recently reported state-of-the-art materials. The CMS membranes derived from PIM showed excellent separation performance and processability due to their high free volume and undetectable glass transition temperature (T_g_), conditions originated from inefficient packing of chains and a rigid backbone. The CMS membranes derived from various PIM precursors (such as PIM-1, PIM-6FDA, and PIM-cyclodextrin) showed high ethylene/ethane separation performance of 10–44 barrer, and 6.29–17.9 for ethylene permeability and ethylene/ethane selectivity, respectively [33,34,35]. However, these polymer precursors are difficult to commercialize in that the material price is high, even though they showed excellent performance for olefin/paraffin separation. Therefore, the CMS membranes derived from cost-effective polymers should be further investigated. 

On the other hand, the pyrolysis process is a key parameter for controlling the pore size structure of the CMS membrane, and for obtaining a desirable separation performance. During pyrolysis, the volatile compounds (e.g., heteroatoms) of the polymer precursor are decomposed, and the molecular structure is rearranged to form an amorphous and rigid carbon structure [36]. Therefore, a variety of polymer precursors can give rise to different carbon microstructures and separation performance even under the same pyrolysis conditions. Furthermore, the selection of a polymer precursor and the optimization of the pyrolysis process are very important for achieving desirable properties and separation performance of a CMS membrane [37]. To optimize the separation performance of CMS membranes derived from various polymer precursors for olefin/paraffin separation, many researchers have studied several effective pyrolysis factors, such as heating rate, soaking time, pyrolysis temperature, and gas atmosphere. 

For the preparation of the CMS membrane, the pyrolysis can be carried out in various temperatures, which is the range for decomposition of the polymer precursor. A higher temperature typically results in lower permeability and higher selectivity, contributing to the greater compactness of the graphitic carbon layers [37,38]. The heating rate and soaking time determine the decomposition rate of the polymer precursor. Therefore, if the soaking time decreases or the heating rate increases, the pore size can be larger. However, a heating rate that is too fast may give rise to defects such as cracks and pinholes. On the other hand, the ambient gases used for pyrolysis can be oxygen-free gases (e.g., He, N_2_, Ar, or vacuum) to avoid undesirable damage [19]. Such pyrolysis of a polymer precursor under inert gas can induce a more open pore structure in the CMS membrane than in the vacuum, due to the higher heat and greater mass transfer. 

In this study, CMS membranes were prepared with a PAN precursor for ethylene/ethane separation. If a CMS membrane derived from a PAN precursor could achieve useful performance for ethylene/ethane separation, its commercialization would be more favorable than with other polymer precursors due to its low cost. Furthermore, the thermosetting PAN polymer precursor shows high thermal stability and high carbon yield. To provide these excellent thermal properties to the PAN precursor, the stabilization as a pre-treatment process should be further carried out. During the stabilization, a cyclization reaction of nitrile groups occurs; this is not common for other polymer precursors, because they already have high thermal stability on their own. Therefore, tuning the stabilization condition is very important to successfully prepare the CMS membrane derived from PAN precursor. Nevertheless, studies on CMS membranes derived from a PAN precursor have hardly been reported. In particular, there are no results on olefin/paraffin separation. Therefore, to optimize the ethylene/ethane separation performance of a PAN-based CMS membrane, we finely tuned and optimized the stabilization and pyrolysis processes in terms of the stabilization temperature and time, as well as the pyrolysis temperature, soaking time, and heating rate. In addition, a thin and uniform PAN layer was formed on an alumina support to provide greater mechanical strength and better permeability.

## 2. Experimental

### 2.1. Materials

PAN copolymer (91.4% acrylonitrile and 8.6% methyl acrylate, Mw = 50,000 g/mol) and N-methyl-2-pyrrolidone (NMP, 99.0%) were purchased from Taekwang Industrial Co. and Duksan Pure Chemicals Co. (Ansan, Korea), respectively. High purity alumina powder (CR15, Baikowski, Poisy, France) was employed to prepare an alumina support disc. For synthesizing Boehmite sol, aluminum tri-sec-butoxide (97%, Acros Organics, Geel, Belgium) and nitric acid (60%, Junsei, Tokyo, Japan) were used. Ethanol (99.5%) was obtained from Duksan Pure Chemicals Co. (Ansan, Korea) All agents above were used without further purification. The helium (99.9999%), argon (99.9999%), and ethylene/ethane mixture (80/20 mol%) were supplied by Joongang Industrial Gas Co. (Daejeon, Korea).

### 2.2. Preparation of PAN-Based CMS Membranes

The PAN-based CMS membrane was prepared on an alumina disc obtained by the pressing method. A portion (1.5 g) of α-alumina powder was put in a disc mold and pressed at 100 barg for 5 s. The alumina disc was then calcined according to the following protocol: (1) increase to 600 °C at a rate of 2 °C/min; (2) maintain the temperature for 1 h; (3) increase the temperature to 1220 °C at the rate of 2.21 °C/min; (4) maintain the temperature for 1 h; (5) allow the disc to cool naturally to the ambient temperature. The thickness and diameter of the resulting disc were 2 and 20 mm, respectively. The surface of the alumina disc was polished and dried prior to coating with an intermediate alumina layer.

To coat the intermediate alumina layer onto the alumina disc, the boehmite sol was synthesized by a sol-gel method reported in the literature [39]. The boehmite sol was diluted with ethanol in the volume ratio of 1:14 (sol:ethanol). The intermediate alumina layer was coated by dipping the surface of the alumina disc into the diluted boehmite sol solution for 20 s. The sol-coated alumina disc was dried in a desiccator at room temperature for more than 3 h. Then, the alumina disc was calcinated at 1000 °C for 1 h while heating at 2.04 °C/min.

**Figure 1:**
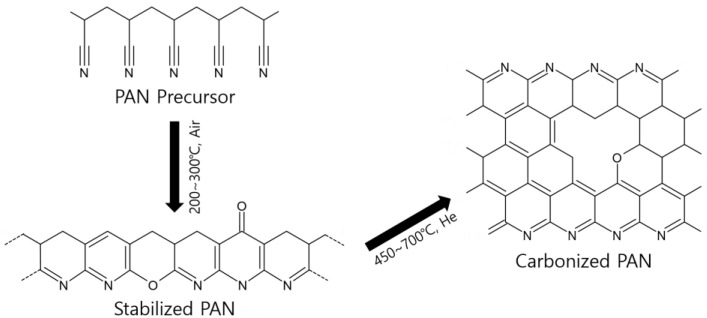
Illustration of the PAN stabilization and carbonization reaction.

To coat the thin PAN polymer layer on the alumina disc with an alumina intermediate layer, the PAN polymer solution was prepared by dissolving 2.5 wt% PAN powder into NMP at 50 °C. The polymer solution was then passed through a syringe filter with a pore size of 5 μm. The PAN polymer layer was formed by a dipping process in the same manner as that used for the boehmite solution. The alumina disc was dipped in the polymer solution for 20 s, and the excess solution was thoroughly removed. The polymer-coated alumina disc was dried in an oven at 60 °C for more than 2 h. Prior to carbonization of the polymer, the stabilization of PAN was carried out to provide improved thermal stability. The dried disc was thermally annealed at the temperature of 200–300 °C for 1–5 h in the air. The stabilization process of the PAN precursor was illustrated in Figure 1. 

After the stabilization process, the PAN-coated alumina disc was carbonized in a tubular furnace under helium gas. Ultra-high purity helium (>99.9999%) was used to purge the furnace at a flow rate of over 1000 cm^3^/min for 10 min. Then, the helium flow rate was set to 50 cm^3^/min during pyrolysis. A low oxygen concentration (<1.0 ppm) was ensured using an oxygen analyzer (Rapidox 2100, Cambridge Sensotec). The pyrolysis conditions were varied in terms of the heating rate (2.94–17.66 °C/min), pyrolysis temperature (450–700 °C), and soaking time (0–5 h). After terminating the pyrolysis process, the CMS membrane was naturally cooled in the tubular furnace to at least 50 °C. The CMS membrane was tested within 10 min of taking it out of the furnace to minimize any unexpected aging of the CMS membrane in the air.

### 2.3. Ethylene/Ethane Mixed Gas Permeation Test

**Figure 2:**
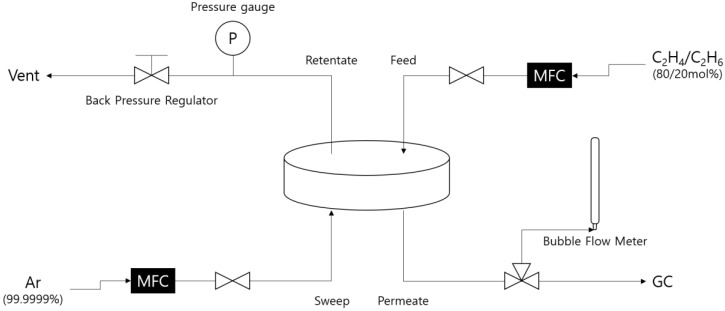
Gas permeation test setup.

Figure 2 shows a gas permeation measurement setup of the CMS composite membrane for the separation of ethylene and ethane in a gas mixture. The 80/20 mol% ethylene/ethane gas mixture was fed onto the surface of the CMS membrane at room temperature, and the stage cut was always maintained at < 0.1 using a mass flow meter (MFC, Brooks instrument 5853E, 5831E). The pressure was adjusted by a back-pressure regulator on the retentate side at 6 barg. A sweeping gas of ultra-high purity argon was provided to the permeate side at the flow rate of 7.2 cm^3^/min. The feed and sweep gases were delivered in a cross-flow. The permeated gas content and flow rate were measured using a gas chromatograph (GC, DS Science iGC 7200A) with a flame ionization detector (FID) and a bubble flow meter, respectively. 

The gas permeance was calculated as below:

(1) $$P_{i}=\frac{x_{p}}{A\left(x_{f}p_{f}-x_{p}p_{p}\right)}\times \frac{p_{atm}}{76}\times \frac{dV}{dt}$$

where $P_{i}$ is the gas permeance of component *i* in gas permeation units (GPU). The terms $x_{f}$, and $x_{p}$ are the mole fraction of component *i* on the feed side and permeate side, respectively. Here, $p_{f}$ and $p_{p}$ are the feed and permeate pressure, respectively. The term A is the effective surface area of the membrane in cm^2^. The atmospheric pressure,$\;p_{atm}$, is the atmospheric pressure in cmHg, and dV/dt is the volumetric flow rate in cm^3^/s.

The selectivity (α) of the mixture gas was calculated as below:

(2) $$\alpha =\frac{P_{i}}{P_{j}}$$

where $P_{i}$ and $P_{j}$ are the gas permeance of ethylene and ethane, respectively.

The experimental data of more than 5 points was obtained after 2 h of gas permeation, then the value was averaged. The gas permeation tests were repeated 3 times, and the standard deviation was calculated.

### 2.4. Characterizations

To examine the cross-sectional morphology of the CMS composite membrane, environmental scanning electron microscopy (ESEM, Thermo Fisher Scientific Quattro S, Waltham, United States) was used. The functional groups of the polymer and CMS membranes were analyzed using Attenuated Total Reflectance Fourier Transform Infrared Spectroscopy (ATR-FTIR, Nicolet 5700, Waltham, United States). Raman spectra was obtained using a SENTERRA spectrometer (Bruker, Billerica, United States) with an excitation source of 532 nm wavelength. A Thermogravimetric Analyzer (TGA, Mettler Toledo TGA/DSC 3+, Columbus, United States) was employed under inert nitrogen gas to investigate the thermal properties of the stabilized PAN precursors. An X-ray Photoelectron Spectrometer (XPS, Kratos AXIS Nova, Wharfside, United Kingdom, 15 KeV accelerating voltage of monochromatic Al Kα X-ray source) was applied for the chemical analysis of the CMS membrane. X-ray diffraction (XRD) patterns were observed using a Rigaku D/max-2200V diffractometer, Tokyo, Japan, and the d-spacing value of the CMS membrane was calculated by Bragg’s law (nλ=2dsinθ, λ: wavelength, d: lattice spacing, θ: diffraction angle). The pore size distribution of the CMS membrane was calculated using the non-local density functional theory method assuming a slit pose geometry with the fitting parameter of Tikhonov Regularization based on CO_2_ adsorption measurements (Microtrac Belsorp Max II, Osaka, Japan). The temperature and relative pressure range ($p/p_{0}$) were 25 °C and 0–1.0, respectively. For analysis of the ATR-FTIR, Raman, XPS, XRD, and CO_2_ adsorption, CMS powder was used instead of the CMS composite membrane due to the analysis limitation imposed by the thin carbon layer. For the preparation of CMS powder, the PAN film was cast with 20 wt% PAN polymer solution in NMP. The dried PAN film was stabilized and carbonized in the same manner. The stabilization time was set to 3 h, and the pyrolysis heating rate and soaking time were fixed to 8.33 °C/min and 1 h, respectively. The CMS film was then finely ground using an agate mortar. 

## 3. Results and Discussion

### 3.1. Structure of CMS Composite Membranes

**Figure 3:**
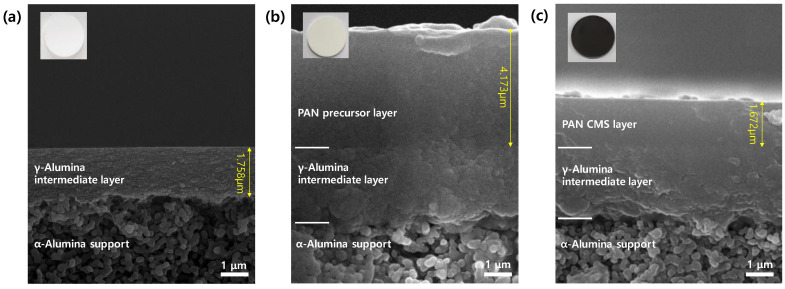
Cross-sectional SEM images of (**a**) alumina intermediate layer-coated membrane, (**b**) PAN polymer-coated membrane, and (**c**) CMS composite membrane (stabilization: 250 °C (temperature), and 1 h (soaking time); pyrolysis: 550 °C (temperature), 8.33 °C/min (heating rate), and 1 h (soaking time).

Figure 3 shows the cross-sectional SEM images of the CMS composite membrane prepared on the alumina disc. The composite structure can provide both high gas permeance and mechanical strength from its thin active layer and rigid support, respectively. Furthermore, the alumina intermediate layer with a smaller pore size was employed to prevent the flow of polymer solution into the mesoporous alumina support. 

The thickness of the alumina intermediate, polymer, and CMS layers was 1.7, 4.1, and 1.6 μm, respectively. After carbonization, the thickness of the CMS layer decreased more than twice compared with that of the PAN layer due to the decomposition of volatile compounds during pyrolysis. The CMS membrane showed high reproducibility without defects, such as cracks or delamination from the alumina support. 

### 3.2. Characterization of the Pristine, Stabilized, and Carbonized PAN

**Figure 4:**
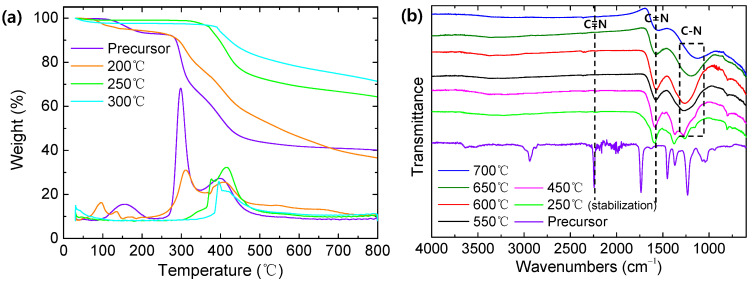
(**a**) TGA analysis and of stabilized PAN and (**b**) ATR-FTIR analysis of the pristine, stabilized PAN, and CMS.

Figure 4a shows the results of TGA analysis for the pristine and stabilized PAN polymers at the temperature of 200, 250, and 300 °C. During the stabilization process, the PAN precursor undergoes three consecutive reactions: (1) cyclization, (2) dehydrogenation, and (3) oxidation. PAN stabilized under air has a better thermal stability than PAN stabilized under O_2_-free gases [40]. Therefore, in this study, the PAN precursor was stabilized under air. The weight loss of the pristine and stabilized PAN polymer at 200 °C was initiated near 250 °C, whereas those stabilized at 250 and 300 °C were decomposed from around 350 and 400 °C. This shows that the temperature of 200 °C may lead to an incomplete stabilization reaction, which is consistent with previously reported results [41,42]. Nevertheless, the stabilized PAN at 200 °C showed a better thermal stability compared with the pristine one. In particular, the stabilized PAN polymers at 250 and 300 °C showed significantly less weight loss at the temperature of 800 °C than that at 200 °C. The higher stabilization temperature improved the thermal stability of the PAN polymer, and contributed to more cyclization reactions between nitrile groups. As such, to impart a suitable thermal property to the PAN polymer, the stabilization process was essential prior to the pyrolysis—unlike other polymers with high thermal stability—and the temperature should be higher than 250 °C.

Figure 4b presents the FTIR analysis results of pristine and stabilized PAN precursors at 250 °C and carbonized PAN at 450–700 °C. For the PAN precursor, strong peaks at 2243, 1732, 1230, and 1032cm^−1^ were assigned to the nitrile, carbonyl, and ether groups, respectively [43]. However, these peaks are not visible after stabilization or carbonization. Instead, the stabilized PAN and carbonized PAN at 450 °C showed C=N (1590 cm^−1^), C-H (1378 cm^−1^), and C-O (1270 cm^−1^) peaks. While increasing the pyrolysis temperature to higher than 550 °C, C-H, and C-O bonds became weak, whereas broad peaks appeared at 1130–1300 cm^−1^ (attributed to C-N stretching). During the stabilization process, nitrile groups were converted to C=N bonds that formed a ladder structure polymer, as shown in Figure 1. Then, the C=N bonds in the carbonized PAN gradually decreased at the higher pyrolysis temperature, while the peak intensity assigned to the C-N bonds became stronger. Finally, the C-N bonds also decreased from 650 °C due to severe decomposition. 

**Figure 5:**
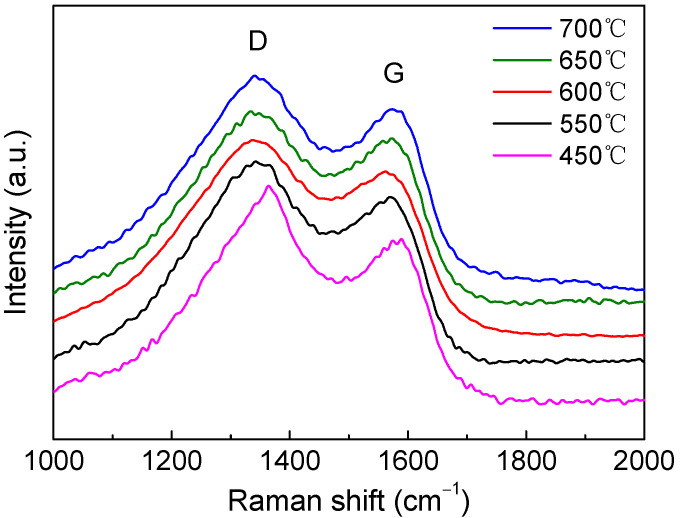
Raman spectra of CMS pyrolyzed at various temperatures.

Figure 5 is the Raman spectra of carbonized PAN precursors. Raman spectra can offer the structural information of carbon-based materials. In general, the amorphous carbon structure shows two broad peaks. A highly oriented graphitic structure appeared at a peak of 1560 cm^−1^, whereas the disordered carbon structure was assigned to 1350 cm^−1^ (the G-band and D-band, respectively) [44]. For the carbonized PAN, herein, two peaks at 1350 and 1580 cm^−1^ were observed, indicating an amorphous structure. Table 1 shows the intensity ratio of the D-band and G-band. With increasing pyrolysis temperature, the I_D_/I_G_ value decreased, implying that the sp^2^-hybridized graphitic carbon became more abundant [45].

**Figure 6:**
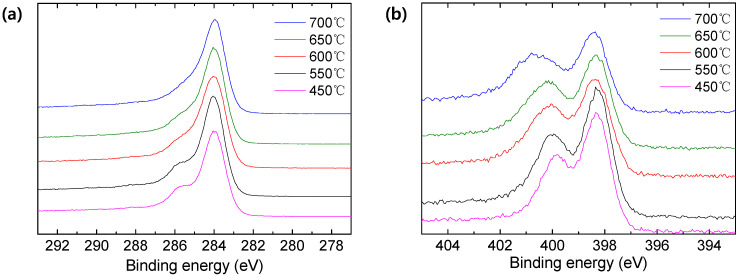
XPS spectra of CMS pyrolyzed at various temperatures (**a**) C1s and (**b**) N1s.

Figure 6 indicates the XPS spectra of CMS prepared at 450–700 °C. The C1s plot in Figure 6a includes four peaks (C-C (sp^2^) at 284.1 eV; C-C (sp^3^) at 284.8 eV; C-O/C-N at 285.8 eV; and C=O/C=N at 287.6 eV) [46]. The CMS showed high ratio of sp^2^ carbon even at low pyrolysis temperature, compared with the sp^3^ carbon due to the cyclization reaction and the aromatization during the stabilization and pyrolysis processes, respectively (Figure S1a). Increasing the pyrolysis temperature decreased the intensity of the C-N and C-O peaks due to the severe decomposition that occurred at higher temperatures, while increasing the sp^2^ carbon. 

On the other hand, the N1s curve contained the peaks of pyridinic N (398.3 eV), pyrrolic N (399.7 eV), and graphitic N (401 eV) [47,48], as shown in Figure 6b. The CMS prepared at 550 °C showed the highest intensity for both pyridinic N and pyrrolic N. This corresponded to the result of FTIR analysis in Figure 4b, where some volatile groups were still observed after pyrolysis at 450 °C. These were then gradually decomposed at temperatures higher than 550 °C. The peak intensity of the N 1s curve decreased at the higher pyrolysis temperature. In addition, for the CMS prepared at 600–700 °C, a new broad peak was observed at 401–402 eV, resulting in the development of graphitic N. Indeed, the graphitic N increased with the increase in pyrolysis temperature, whereas the pyridinic N and pyrrolic N decreased (Figure S1b). In particular, the severe decomposition of pyrrolic N was observed. The CMS was transformed to more graphitic carbon structure at the higher temperature, which corresponded to the results of Raman analysis. 

XPS elemental analysis was carried out to investigate the components of CMS after pyrolysis, as indicated in Table 2. The initial nitrogen ratio of CMS pyrolyzed at 450 °C was 13.73%, which then gradually decreased with the increase in the pyrolysis temperature. On the contrary, the carbon ratio increased due to the decomposition of volatile compounds containing nitrogen and oxygen and an evolution of heteroatoms. On the other hand, even though the oxygen composition also decreased with increase in the pyrolysis temperature, the highest oxygen content was observed from the CMS pyrolyzed at 600 °C. This might be attributed to the excess oxygen chemisorption during the exposure to air for sample preparation and storage. Thus, it is difficult to give meaningful discussion with the oxygen contents, because the oxygen content of the CMS membrane was varied depending on the exposure conditions in the air.

### 3.3. Ethylene/Ethane Mixed Gas Separation Performance of CMS Membranes

The permeation test of ethylene/ethane mixed gas was performed with the CMS membranes prepared under a variety of stabilization and pyrolysis conditions. For the permeation test, the ethylene/ethane mixture gas ratio was 80/20 mol%, and the pressure and temperature were set to 6 barg and room temperature, respectively. 

**Figure 7:**
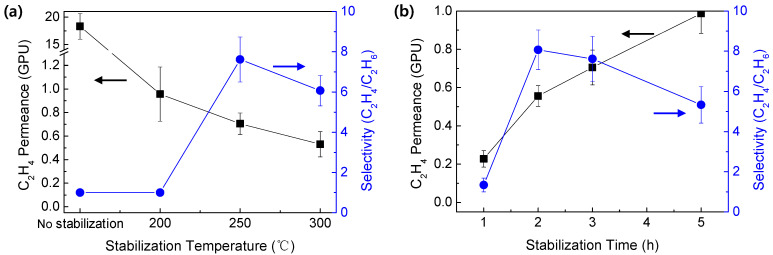
Ethylene/ethane mixed gas separation performance of CMS membranes prepared in a variety of (**a**) stabilization temperature (stabilization time: 3 h) and (**b**) stabilization time (stabilization temperature: 250 °C).

Figure 7 indicates the ethylene/ethane mixed gas separation performance of CMS membranes prepared from the PAN precursor at different stabilization temperatures and times. For this test, the pyrolysis temperature, heating rate, and soaking time were fixed to 550 °C, 8.83 °C/min, and 1 h, respectively. The separation performance of CMS membranes stabilized at different temperatures is shown in Figure 7a. The CMS membranes prepared without the stabilization and with the stabilization at 200 °C showed no ethylene/ethane selectivity with relatively high ethylene permeance of 18.3 and 0.96 GPU, respectively. This is attributed to its low thermal stability, as shown in the results of the TGA analysis (Figure 3), leading to severe decomposition and thereby a structure of large pores. In addition, the low thermal stability of the polymer precursor can give rise to low carbon yield, in which case it is unable to form the selective molecular sieving pores needed for ethylene/ethane separation. The most useful separation performance (ethylene permeance of 0.71 GPU and ethylene/ethane selectivity of 7.62) was observed with the CMS membrane stabilized at 250 °C. It was considered that the stabilization at 250 °C provided sufficient thermal stability to form the ethylene/ethane selective pore structure. However, the higher stabilization temperature of 300 °C lowered the separation performance. This may be attributed to the excessively improved thermal stability of the PAN precursor during the stabilization process, which can reduce the pore size and porosity due to insufficient rearrangement of carbon layers during carbonization. 

Figure 7b shows the mixed gas separation performance of CMS membranes prepared with different stabilization times. The stabilization temperature was set to 250 °C. Increasing the stabilization time resulted in the increase of gas permeance. With increase in the stabilization time, more oxygen can react with the PAN precursor during the stabilization process. The oxygen atoms may hinder aromatization reactions during carbonization. Consequently, a less dense carbon structure was obtained after pyrolysis, leading to enlargement of the pore size. However, the CMS membrane derived from the PAN precursor stabilized for 1 h showed no ethylene/ethane separation property, whereas the useful ethylene/ethane selectivity was observed at stabilization times of more than 2 h. In particular, the highest ethylene/ethane selectivity could be obtained with stabilization for 2 h. If the stabilization time was not enough for the oxidation reaction, an excessively dense structure was formed due to the absence of oxygen atoms. Therefore, few selective pores were formed, meaning that both ethylene and ethane were rejected. If the stabilization time increased, the pore size enlarged due to more oxidation, and the selective pore structure was formed. However, using an excessive stabilization time may induce pores so large that ethane can penetrate them, resulting in the reduction of the ethylene/ethane selectivity. In a further separation test of CMS membranes prepared under various pyrolysis conditions, the stabilization temperature and time were fixed to 250 °C and 3 h. 

**Figure 8:**
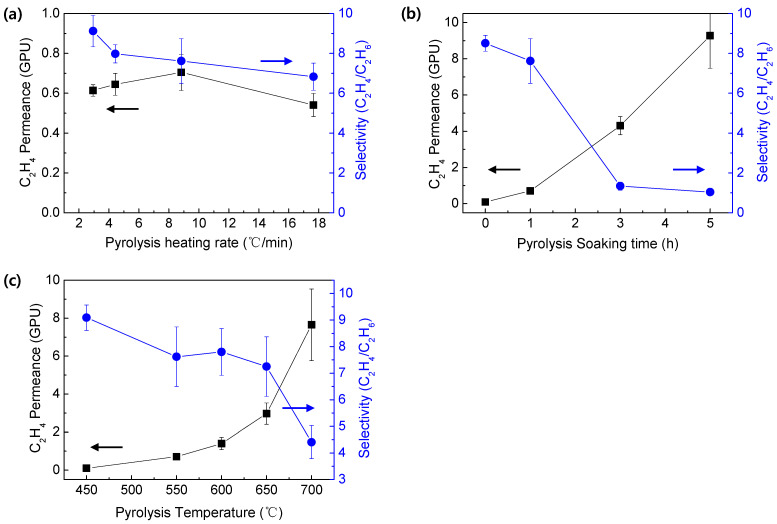
Ethylene/ethane mixed gas separation performance of CMS membranes prepared under various (**a**) pyrolysis heating rates (soaking time: 1 h, pyrolysis temperature: 550 °C), (**b**) pyrolysis soaking time (heating rate: 8.33 °C/min, pyrolysis temperature: 550 °C), and (**c**) pyrolysis temperature (heating rate: 8.33 °C/min, soaking time: 1 h).

Figure 8 shows the effect of pyrolysis parameters including temperature, soaking time, and heating rate on the separation performance of a CMS membrane. The ethylene/ethane separation performance of a CMS membrane, according to the pyrolysis heating rate, is indicated in Figure 8a. The soaking time and pyrolysis temperature were fixed to 1 h and 550 °C, respectively. In general, it has been reported that a high heating rate may cause imperfect pore structure (such as pinholes), whereas a low heating rate forms small pores [49,50]. Therefore, herein, excellent ethylene/ethane selectivity was observed with a heating rate of 2.29 °C/min. However, it should be noted that additional cyclization reaction can occur in the low temperature range (200–300 °C) during pyrolysis. In addition, this can intensify at the lower heating rate due to the longer retention time at the reactive temperature. Thus, further cyclization of the PAN precursor can give rise to a denser carbon structure, resulting in lower gas permeance. Nevertheless, the heating rate had relatively less effect on the separation performance than the other parameters did. 

Figure 8b indicates the separation performance of CMS membranes prepared with different soaking times during pyrolysis. The heating rate and pyrolysis temperature were set to 8.33 °C/min and 550 °C, respectively. With an increase in the soaking time, the ethylene permeance increased, whereas the ethylene/ethane selectivity decreased to ~1. This means that the higher soaking time led to a larger pore structure in the CMS membrane. A similar trend was observed in the CMS membranes prepared at various pyrolysis temperatures (Figure 8c). A longer soaking time and higher pyrolysis temperature generally cause a smaller pore structure due to the microstructural rearrangement and compaction of carbon layers, resulting in improved selectivity, unlike the results in this study [38,51]. The PAN precursor uncommonly undergoes cyclization and aromatization at a relatively low temperature, leading to a highly dense structure. However, with the increase in the soaking time or pyrolysis temperature, the chains in the dense layers can be decomposed. Therefore, the gas permeance could be increased at the higher pyrolysis temperatures and soaking times. This unusual phenomenon has sometimes been reported in the literature. A.F. Ismail prepared a CMS membrane derived from a PAN precursor, which showed the increase in the pore size with the increase in the pyrolysis temperature [16]. It was attributed to the expelling of the carbon atoms as carbon monoxide. They also mentioned that the pore structure can vary depending on the nature and morphology of the polymer precursor. In addition, a similar result was reported in the CMS membrane prepared with the cured phenolic resin [21]. In line with increasing the pyrolysis temperature, the gas permeance constantly increased to 700 °C and tended to decrease slightly over 800 °C. In the two literatures, the pores were initially formed and enlarged with an increase in the pyrolysis temperature (~700 °C), but were gradually shrunk from a certain temperature (>800 °C). In our study, if the pyrolysis temperature further increased (>800 °C), the gas permeance would decrease due to the shrinkage of carbon structure. However, the pyrolysis at a higher temperature was undesirable, because the selectivity constantly decreased, as shown in Figure 8c. 

**Figure 9:**
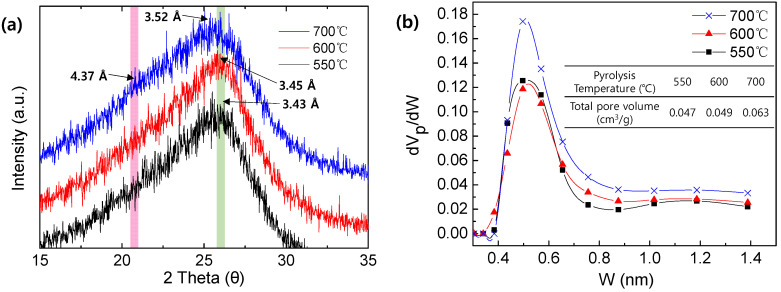
(**a**) XRD pattern and (**b**) pore size distribution of CMS pyrolyzed at 550, 600, and 700 °C.

To prove the effect of pyrolysis temperature on the pore structure of a CMS membrane derived from the PAN precursor, the pore size distribution and interplanar distance of the CMS membranes were investigated via CO_2_ adsorption and XRD analysis, respectively (Figure 9). As the pyrolysis temperature increased, two thetas were slightly shifted to a smaller value, indicating a larger interplanar distance. Moreover, a peak corresponding to d-spacing of 4.37 Å was newly observed in the CMS prepared at 700 °C, indicating that the structure with a large d-spacing was noticeably developed. The d-spacing does not signify the actual pore size of the CMS membrane. Nevertheless, it showed that increasing the pyrolysis temperature leads to a larger pore size of CMS membrane. The pore size distribution obtained by CO_2_ adsorption showed a greater increase in the pore volume for both ultramicropores and micropores at the higher pyrolysis temperature, than for the pore size, which plays a big role in the increase in gas permeance. This simultaneously decreased the selectivity of the CMS membrane due to the volume increase of the non-selective pores larger than 0.5 nm, even though the increase rate of the pore volume in the range of 0.5–1.4 nm was less than that in 0.4–0.5 nm. In addition, for the separation of ethylene and ethane with very similar molecular size, this slight increase of the pore volume might significantly influence the reduction in selectivity. In particular, the interplanar distance and pore volume of CMS membrane pyrolyzed at 700 °C were remarkably increased, which is consistent with the results shown in Figure 8b,c.

### 3.4. Comparison of Ethylene/Ethane Separation Performance 

**Figure 10:**
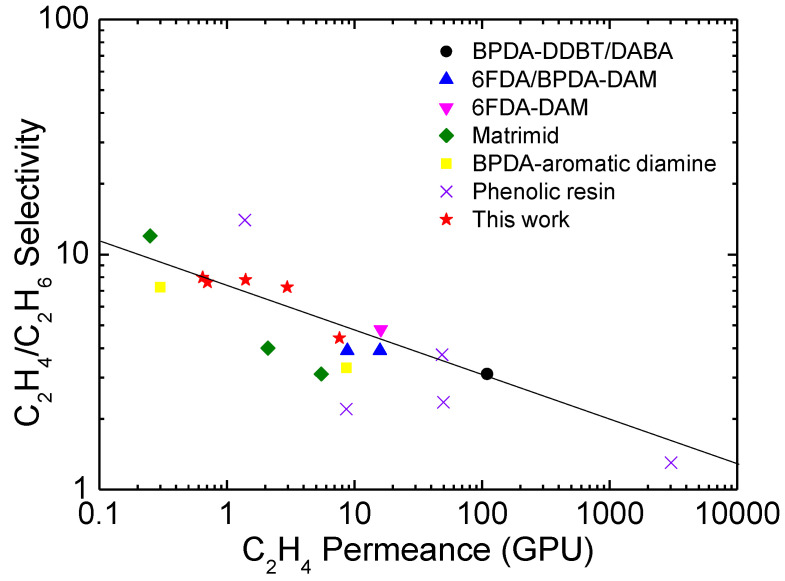
Ethylene/ethane separation theoretical upper bound [9,31,32,52,53,54,55,56,57].

The ethylene/ethane separation performance of CMS membranes derived from the PAN precursor was compared with those prepared with other polymer precursors, such as polyimide and phenolic resin, as shown in Figure 10. The PAN-based CMS membranes prepared in this study surpassed the upper bound trade off. The optimized stabilization temperature and soaking time were 250 °C and 3 h, respectively, and the optimized pyrolysis temperature, soaking time, and heating rate were 600–650 °C, 1 h, and 8.33 °C/min, respectively. These CMS membranes achieved excellent ethylene permeance (1.40–2.97 GPU) and ethylene/ethane selectivity (7.25–7.80). Furthermore, these values were comparable to those of other CMS membranes. 

## 4. Conclusions

For ethylene/ethane separation, a CMS membrane derived from a PAN precursor was successfully prepared on an alumina disc coated with an alumina intermediate layer. To improve the thermal stability of the PAN precursor, a stabilization process was employed prior to pyrolysis, and the stabilization temperature and soaking time were optimized. In addition, the effect of the pyrolysis conditions on the CMS membranes was studied via XPS, FTIR, Raman, XRD, and CO_2_ adsorption. The CMS pyrolyzed at higher temperature possessed a more ordered carbon structure, a larger interplanar distance, and a greater pore volume. These trends were the opposite of those typically reported due to the stabilization process of the PAN precursor.

An ethylene/ethane mixed gas permeation test was carried out with CMS membranes prepared under a variety of stabilization and pyrolysis conditions. The CMS membrane pyrolyzed at higher temperature showed higher ethylene permeance and lower ethylene/ethane selectivity, which was attributed to the larger interplanar distance and pore volume. The lower heating rate can induce further cyclization reactions due to the longer retention time at the reactive temperature. This leads to a denser carbon structure and low gas permeance. The optimized CMS membrane (stabilization temperature: 250 °C, stabilization time: 3 h, pyrolysis temperature: 650 °C, pyrolysis soaking time: 1 h, and pyrolysis heating rate: 8.33 °C/min) derived from the PAN precursor showed excellent ethylene/ethane separation performance (ethylene permeance: 2.97 GPU, and ethylene/ethane selectivity: 7.25), which surpassed the upper bound trade off. 

If considering commercialization of the CMS membrane, the cheaper PAN precursor could be a candidate with useful properties for ethylene/ethane separation in that most of the polymer precursors good for CMS membranes with excellent separation performance were costly. Moreover, the supported CMS membrane can provide high mechanical strength, as well as high gas permeance, which facilitates the scale-up of the CMS membrane due to being easy to handle. The ceramic support with a low material price and a simple manufacturing process can also offer economic efficiency. Nevertheless, the CMS membrane derived from the PAN precursor showed relatively lower ethylene permeance compared with other polymer precursors. Further study is currently in progress to improve the gas permeance of a CMS membrane derived from the PAN precursor.

## Figures and Tables

**Table 1:** Intensity ratio of disordered and graphitic peaks obtained by Raman analysis of CMS pyrolyzed at various temperatures.

Pyrolysis Temperature (°C)	450	550	600	650	700
I_D_/I_G_	1.32	1.22	1.19	1.16	1.10

**Table 2:** XPS elemental analysis of CMS pyrolyzed at a variety of temperature.

Pyrolysis Temperature (°C)	Elemental (At. %)		
	C	N	O
450	80.79	13.73	5.48
550	81.99	13.10	4.91
600	81.65	12.61	5.74
650	83.02	12.29	4.67
700	85.49	11.25	3.26

## Supplementary Materials

The following are available online at https://www.mdpi.com/article/10.3390/membranes12010093/s1, Figure S1: Deconvolution of C1s XPS spectra of CMSs pyrolyzed at different temperature, Figure S2: Deconvolution of N1s XPS spectra of CMSs pyrolyzed at different temperature.
