# Supplementary material for: Tailoring the Stabilization and Pyrolysis Processes of Carbon Molecular Sieve Membrane Derived from Polyacrylonitrile for Ethylene/Ethane Separation
Source: Membranes (Basel). 2022 Jan 15;12(1):93. doi: 10.3390/membranes12010093 (PMC8781130; doi:10.3390/membranes12010093)
Supplement: Supplementary file 1 [file membranes-12-00093-s001.zip › membranes-1540068-supplementary.pdf]

Tailoring the stabilization and pyrolysis processes of carbon molecular sieve membrane derived from polyacrylonitrile for ethylene/ethane separation

DaeHun Kim[a,b], YongSung Kwon[a,c], Jung-Hyun Lee[b], Seong-Joong Kim[d*] and You-In Park[a*]

[a] Green Carbon research Center, Korea Research Institute of Chemical Technology (KRICT), Gajeong-ro 141, Yuseong-gu, Daejeon 34114, Republic of Korea

[b] Department of Chemical and Biological Engineering, Korea University, 5-1 Anam-dong, Seongbuk-gu, Seoul 136-713, Republic of Korea

[c] Department of Chemical and Biomolecular Engineering, Korea Advanced Institute of Science and Technology (KAIST), Daejeon, 34141, Republic of Korea

[d] Center for Convergence Bioceramic Materials, Convergence R&D Division, Korea Institute of Ceramic Engineering and Technology (KICET), 202 Osongsaengmyeong 1-ro, Osong-eup, Heungdeok-gu, Cheongju-si, Chungcheongbuk-do, 28160, Republic of Korea

* Correspondence: authors: sjkim@kicet.re.kr (S.-J. Kim); yipark@krict.re.kr (Y.-I. Park)

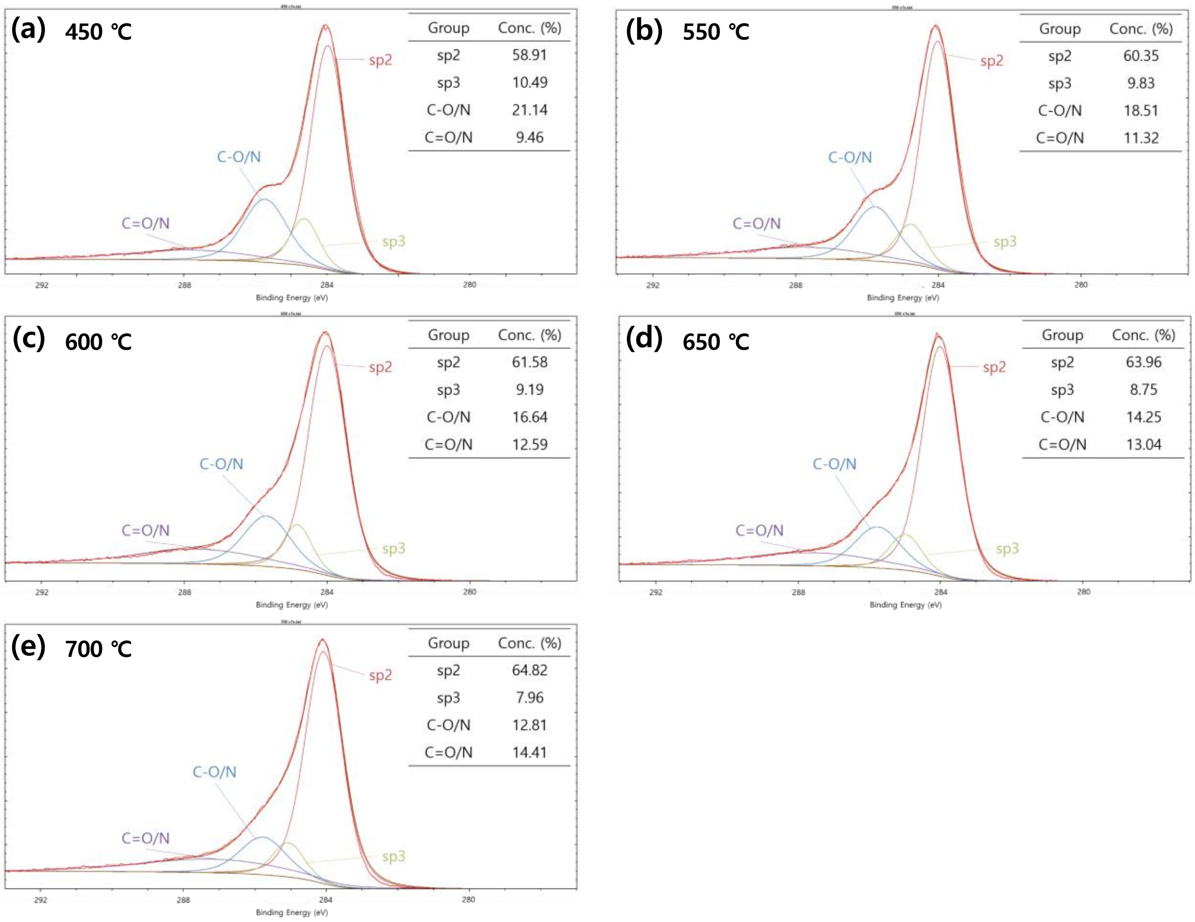

Figure S1. Deconvolution of C1s XPS spectra of CMSs pyrolyzed at different temperature.

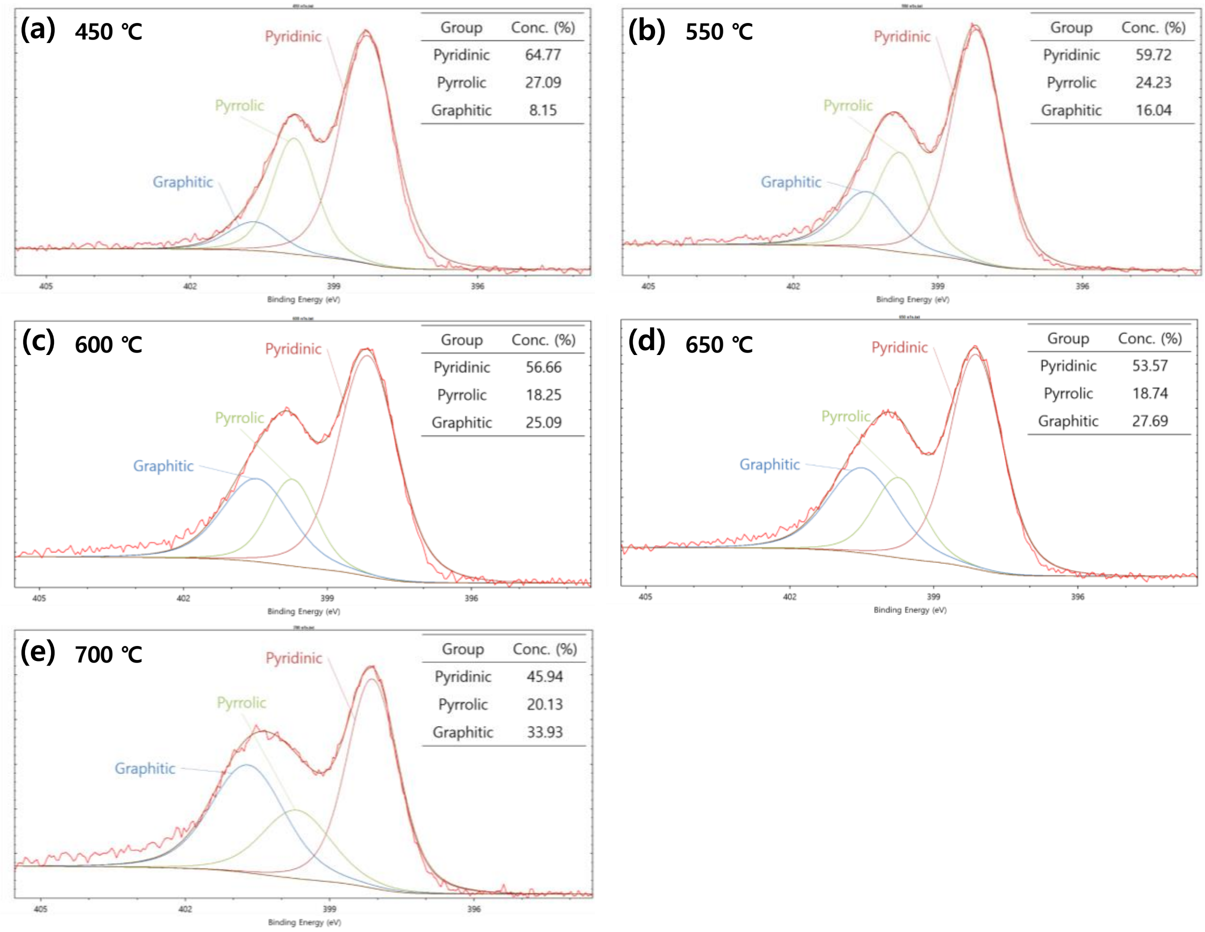

Figure S2. Deconvolution of N1s XPS spectra of CMSs pyrolyzed at different temperature.
